# Feasibility and patient satisfaction with smoking cessation interventions for prevention of healthcare-associated infections in inpatients

**DOI:** 10.1186/s13011-016-0059-0

**Published:** 2016-04-26

**Authors:** Danielle M. Schulte, Megan Duster, Simone Warrack, Susan Valentine, Douglas Jorenby, Daniel Shirley, James Sosman, Sheryl Catz, Nasia Safdar

**Affiliations:** Department of Medicine, University of Wisconsin-Madison School of Medicine and Public Health, Madison, WI USA; Department of Population Health Sciences, University of Wisconsin – Madison School of Medicine and Public Health, Madison, WI USA; Center for Tobacco Research and Intervention, University of Wisconsin-Madison, Madison, WI USA; Betty Irene Moore School of Nursing, University of California Davis, Sacramento, CA USA; University of Wisconsin Hospitals and Clinics, Madison, WI USA; William S. Middleton Memorial Veterans Affairs Medical Center, 5221 MFCB, 1685 Highland Avenue, Madison, WI 53705 USA

**Keywords:** Healthcare-associated infections, *Staphylococcus aureus*, MRSA, Tobacco cessation, Infection control

## Abstract

**Background:**

Smoking increases hospitalization and healthcare-associated infection. Our primary aim of this pilot, randomized-controlled trial was to examine the feasibility and acceptability of a tobacco cessation intervention compared with usual care in inpatients. *S. aureus* carriage, healthcare-associated infections and infections post discharge were exploratory outcomes.

**Methods:**

Current inpatient smokers from a university hospital facility were randomized to usual care or a face to face tobacco cessation counseling session where patients’ tobacco use and strategies for quitting were discussed. Patient engagement, satisfaction and withdrawal symptoms were measured at 1 week and 12 weeks post discharge. Nasal swabs were collected at enrollment and discharge and assessed for *S. aureus* colonization. *P*-values were calculated using Fisher’s exact and *t*-tests were used to compare groups.

**Results:**

For the study’s primary outcome, participants reported the intervention as being generally acceptable and reported high overall levels of satisfaction, with a Likert scale score of at least 4/5 for all measures of satisfaction. No subjects utilized free tobacco cessation services after discharge. 83 % of the intervention group and 93 % of the control group smoked at least one cigarette after discharge. Secondary outcomes with regard to infections showed that, at discharge, 12 % of the intervention group (*n* = 17) and 18 % of the control group (*n* = 22) tested positive for *S. aureus*. After 3 months, 9 % of the intervention group developed infection, 41 % visited an emergency room, and 24 % were readmitted within 3 months post-discharge, compared to 27, 32 and 36 % of the control group respectively.

**Conclusions:**

With regards to the primary aim of this study, there were overall high levels of satisfaction with the intervention, indicating good feasibility and acceptance among patients. However, more intensive interventions in hospitalized patients and impact on healthcare-associated infections and post-discharge infections should be explored.

## Background

Smoking is a major risk factor for many types of infections [1]. Adult smokers are at increased risk of respiratory colonization and infection by several bacterial pathogens, including *Streptococcus pneumonia*, *Neisseria meningitidis*, *Haemophilus influenzae* and *Legionella pneumophila* [2–6]. The nasal flora of smokers has also been shown to contain fewer normal bacteria and more pathogenic organisms, but this imbalance can revert to normal after quitting smoking [2–6]. Direct evidence linking smoking to an increased risk of healthcare-associated infection (HAI) is limited. However, because tobacco use affects an individual’s normal flora [6] and negatively affects the immune system [7, 8], the connection has been established with one study that showed a 75 % increased risk of *Clostridium difficile*-associated diarrhea in smokers compared with non-smokers [9]. HAIs such as *C. difficile* colitis, methicillin-resistant *Staphylococcus aureus* (MRSA) and respiratory infections are of major public health importance; prevention of HAIs has been deemed essential by all major public health agencies [10–12]. Given that smoking increases the risk of HAI, strategies for tobacco cessation may be important in reducing the burden of HAI.

Limited data exist on the utility of smoking cessation interventions for inpatients [13–15] and to our knowledge no study has examined the interaction between smoking cessation and HAI. Hospitals in the United States must have no-smoking policies for accreditation and therefore, a hospital stay requires a smoker to abstain temporarily from tobacco, offering hospitalized smokers an opportunity to initiate cessation. However, this can precipitate nicotine-withdrawal symptoms in hospitalized smokers, causing discomfort and potentially reducing smokers’ adherence to no-smoking policies. Inpatient nicotine-replacement therapy reduces the symptoms of nicotine withdrawal and could increase the chance of remaining abstinent from tobacco after discharge. In fact, the Agency for Healthcare Research and Quality recommends that all hospitalized smokers be offered all effective smoking-cessation treatments and medications as outlined in the 2008 Clinical Practice Guideline [16]. However, there is great variability in the extent to which this is followed [17].

We undertook a pilot randomized-controlled trial at a tertiary care teaching hospital with the primary aim of examining the feasibility and acceptability of implementation of a brief intensive smoking cessation intervention for inpatient smokers compared with usual care. Accordingly, we examined patient satisfaction with the intervention methods as well as patient engagement and withdrawal symptoms, and, as an exploratory outcome, measured HAI and post discharge infections up to 3 months. We hypothesized that our proposed intervention to encourage tobacco cessation would be feasible and associated with high patient satisfaction.

## Methods

**Fig. 1:**
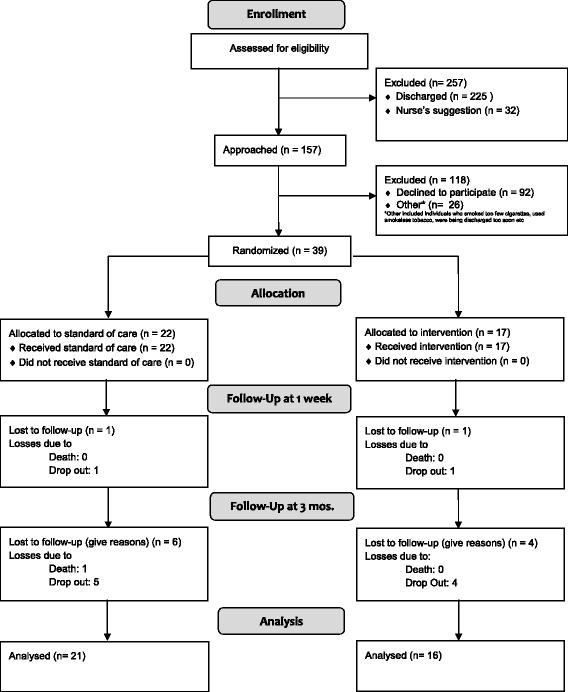
Recruitment Consort Diagram

A two-armed parallel design randomized trial was performed, comparing the feasibility of a tobacco cessation intervention with usual care smoking cessation in hospitalized patients. Potential participants from the University of Wisconsin Hospital & Clinics inpatient units were identified by research team members using daily admission lists at the hospital. Current smokers (who smoked greater than 9 cigarettes per day on average for last 6 months prior to admission, and who also had an anticipated hospital stay of at least 3 days) aged 18 or older were identified from the electronic medical record. Patients admitted to the hospital within the last 24 h were approached for participation by the research team and were evaluated for their desire to quit smoking. Only patients with a motivation to quit were consented, enrolled, and randomized to receive either the study “intervention” or “usual care” for smoking cessation (Fig. 1). Randomization was achieved using a computer-generated randomization table created by the research team. The research team were aware of the subjects’ randomization allocation at enrollment and follow-up so as to allow the appropriate intervention and follow-up questionnaire to be administered. Subjects were not made directly aware of their allocation, however due to differences in the cessation interventions, blinding was unable to be assured. Patients in the intensive care, neurology and psychiatric units were excluded from inclusion in the study as well as those prohibited by their nurses or doctors from participating. The study protocol was approved by the Institutional Review Board at the University of Wisconsin School of Medicine and Public Health.

### Usual care

Usual care at our institution consisted of an assessment of tobacco use at admission, followed by a brief counseling session provided by the treating team during hospitalization, including evidence-based pharmacotherapy interventions such as nicotine patches, gum and lozenges, bupriopion and varencicline as applicable. The usual care counseling session was meant to educate patients on strategies for smoking cessation, and provide patients with informational and educational materials regarding smoking cessation.

### Intervention

The study intervention consisted of a brief counseling session [18] provided by a research team member, trained in patient cessation methods and procedures, and introduction to the Wisconsin Quit Line. The Quit Line is a free and confidential smoking cessation resource that helps smokers create individualized tobacco cessation plans and offers free materials, medication and referrals to local tobacco cessation resources and services [19].

Lasting 15 – 20 min, the trained researcher assessed subject readiness to quit, which consisted of open response self-report of the desire to quit, focused on individualized strategies for smoking cessation and provided patients with intervention-specific informational materials, including information on how to contact the Wisconsin Quit Line.

### Microbiology methods

For all subjects, a nasal swab was collected upon study enrollment and upon hospital discharge to assess baseline *S. aureus* colonization. Specimens were enriched in broth and cultured on mannitol salt agar containing cefoxitin (4 micrograms/mL) for 48 h. Catalase-positive, coagulase-positive, Gram positive cocci were confirmed as MRSA using the Kirby Bauer disc diffusion method. Swabs were not obtained at any subsequent ER or hospital visits as subjects were not followed in person after hospital discharge.

### Definition of Infections

We used the Center for Disease Control and Prevention’s definition of healthcare-associated infection defined as meeting criteria for infection for a specific body site on after the 3^rd^ calendar day of hospitalization [20].

### Follow-up procedures

Post-discharge from the hospital, a follow-up phone call was made at two intervals, 2–7 days post-discharge and again at 12 weeks post-discharge by a trained research staff member. These calls were used to gauge subject engagement and identify any tobacco withdrawal symptoms. During each call, staff conducted a relapse assessment, obtained information on utilization of smoking cessation treatments (i.e., Wisconsin Tobacco Quit Line, nicotine patch, etc.) and conducted withdrawal assessment measures using validated measures of affect and nicotine withdrawal (Positive and Negative Affect Schedule [PANAS] and Wisconsin Smoking Withdrawal Scale [WSWS]) [21, 22]. Staff also utilized a self-efficiency assessment that consisted of a question regarding subjects’ expectations on whether they would be a smoker in the future. In the intervention group, patient perceptions of the acceptability and satisfaction with the study intervention using a 5-point Likert scale and data on infection incidence and readmissions using a standardized questionnaire were also obtained. The answers for the 5-point Likert scale spanned values from 1–5, with 1 representing “Not Satisfied at All” and 5 representing “Extremely Satisfied”, and measured patient perceptions of the intervention content, length, and timing, the study team, physician support and family support. Relapse assessment and utilization of smoking cessation treatments were measured as occurring since the previous contact with research staff. HAI incidence and readmissions were measured as occurring any time since discharge. Subjects were also given an open-ended question, allowing for any additional comments concerning the study procedures to be made. Due to the nature of these assessments, researchers were not able to be blinded to participants’ study group.

### Statistical analysis

Mean group scores were calculated for each follow-up measure. Subjects’ baseline characteristics and follow-up scores were compared using Fisher’s exact tests and *t*-tests for categorical and continuous variables respectively in SAS 9.4 software (SAS Institute Inc., Cary NC USA). A *p*-value of < 0.05 was considered statistically significant. Subjects with missing data were excluded from analysis.

## Results

Thirty-nine subjects enrolled in the study. Twenty-two subjects were randomized to the usual care group and 17 were randomized to the intervention group (Fig. 1). Age was the only baseline feature of the groups to differ significantly, with those in the intervention group being older (Table 1). Seven individuals from the usual care group and two individuals from the intervention group did not have a discharge swab collected because they were discharged from the hospital before swabs could be obtained. After 1 week, 2 subjects were lost to follow-up leaving 16 subjects in the intervention arm and 21 subjects in the usual care arm. After 3 months, 10 more subjects were lost to follow-up leaving 12 subjects in the intervention arm and 15 subjects in the usual care arm.

**Table 1:** Baseline characteristics

	Intervention (*n* = 17)	Usual care (*n* = 22)	*P*-value*
Mean age, years (Standard deviation)	53.5 (12.5)	44.7 (12.8)	0.039
Gender			1.000
Males (%)	9 (52.9 %)	12 (54.5 %)	
Females (%)	8 (47.1 %)	10 (45.5 %)	
Race			0.634
White/Caucasian (%)	12 (70.6 %)	17(77.3 %)	
Black/African Am. (%)	2 (11.8 %)	2 (9.1 %)	
Other^a^	1(5.9 %)	1(4.5 %)	
Not Reported (%)	2 (11.8 %)	2 (9.1 %)	
Ethnicity			1.000
Hispanic (%)	0	1 (4.5 %)	
Non-Hispanic (%)	15 (88.2 %)	17 (77.3 %)	
Not Reported (%)	2 (11.8 %)	4 (18.2 %)	
Colonization			
MSSA at baseline	0	6 (27.3 %)	0.063
MRSA at baseline	2 (11.8 %)	0	0.194
MSSA at discharge	1 (7.1 %)	4 (26.7 %)	0.330
MRSA at discharge	1 (6.7 %)	0	1.000
Average cigarettes per day (SD)	17.6 (9.0)	16.5 (11.5)	0.944
Infection history			
History of VRE (%)	0	0	-
History of MRSA (%)	0	1(4.5 %)	1.000
History of C. difficile (%)	0	0	-
Hosp history			
Hospitalized 3months. prior (%)	2 (11.8 %)	5 (22.7 %)	0.438
Surgery 3months. prior (%)	2 (11.8 %)	3 (13.6 %)	1.000
Immuno comp. status			
Active Cancer (%)	5 (29.4 %)	1 (4.5 %)	0.068
Immunosuppressive Drugs 30 days prior (%)	2 (11.8 %)	2 (9.1 %)	1.000
Diabetes (%)	3 (17.6 %)	6 (27.3 %)	0.704
Comorbidities			
Liver disease (%)	1 (5.9 %)	4 (18.2 %)	0.363
Cardiovascular disease (%)	10 (58.9 %)	10 (45.5 %)	0.523
Respiratory disease (%)	7 (41.2 %)	10 (45.5 %)	1.000
GI disease (%)	6 (35.3 %)	9 (40.9 %)	0.753
Antibiotics			
Antibiotics within 30 days prior to admission (%)	3 (17.6 %)	6 (27.3 %)	0.704
Other risk factors			
Open Wound (%)	4 (23.5 %)	7 (31.8 %)	0.725
Alcohol Use (%)	13 (76.5 %)	13 (59.1 %)	0.456
Drug Use (%)	3 (17.6 %)	4 (18.2 %)	1.000
AVG. length of stay (SD)*	5.2 (2.0)	4.2 (2.8)	0.227

Continuous data were analyzed using *t*-tests and dichotomous data were analyzed using Fisher’s exact tests with significance delineated at *p* <0.05

^a^One subject in the intervention group reported his race to be African American and Native American and one subject from the usual care group reported his race to be “Mexican”

*One subject from the usual care group had a length of stay of 26 days and was excluded from the average calculation

At admission, 27 % of subjects from the usual care group and 0 % of subjects from the intervention group had nasal swabs positive for methicillin-susceptible *Staphylococcus aureus* (MSSA). At admission, 0 % of subjects from the usual care group and 12 % of subjects from the intervention group had nasal swabs positive for methicillin-resistant *Staphylococcus aureus* (MRSA). Among those who had a discharge swab collected, 27 % of subjects from the usual care group and 7 % of subjects from the intervention group had nasal swabs positive for MSSA and 0 % from the usual care group 7 % of subjects from the intervention group had a nasal swab positive for MRSA. One subject was observed to have been negative at admission and became MSSA positive at discharge (Table 1).

There were no statistically different relapse or health outcomes measured at 1 week or 3 months (Table 2). At each follow-up stage, subjects in both groups continued to have high smoking rates. At week one post discharge 68 % of subjects smoked at least one cigarette since discharge. At the 3-month follow-up, 89 % of subjects smoked at least one cigarette since discharge. One subject also reported developing an infection during a hospital stay, and 3 subjects at the one-week time point, and 5 subjects at 3-month follow-up, reported developing an infection post hospital discharge (Table 2). Infections included surgical site infection, urinary tract infection, sinus infection and pneumonia. Subjects also had high rates of emergency room visitation and hospital readmission post discharge. At one week, 10 % of subjects had visited an emergency room, and at 3 months 36 % of subjects had visited an emergency room. Also, at one week 8 % of subjects had been readmitted to a hospital and at 3 months 31 % had been readmitted. None of the subjects reported utilizing the Wisconsin Quit Line to aid in their smoking cessation attempt.

**Table 2:** Relapse and health outcome week 1 and 3 months

	Intervention (*n* = 17)	Usual care (*n* = 22)	Total (*n* = 39)	*P*-value^*^
Outcomes at week 1				
Smoking since DC(%)	11/16 (68.7 %)	14/21 (66.6 %)	25/37 (67.6 %)	1.000
Alcohol (%)	1/16 (6.3 %)	2/21 (9.5 %)	3/37	1.000
Quit Line (%)^a^	0/8	0/12	0/20	-
Other cessation program/Medication Use (%)^a^	2/8 (25.0 %)	0/12	2/20 (1.0 %)	0.164
Infection during stay (%)	0/16	1/21 (4.8 %)	1/37 (2.7 %)	1.000
Infection Post DC (%)	1/16 (6.3 %)	2/21 (9.5 %)	3/37 (8.1 %)	1.00
ER visit (%)^b^	2/17 (11.8 %)	2/22 (9.1 %)	4/39 (10.3 %)	0.568
Hospital readmission (%)^b^	1/17 (5.9 %)	2/22 (9.1 %)	3/39 (7.7 %)	0.496
Outcomes at 3 months				
Smoking in last 7 days (%)	8/12 (66.7 %)	11/15 (73.3 %)	19/27 (70.4 %)	1.000
Smoking at all since DC (%)	10/12 (83.3 %)	14/15 (93.3 %)	24/27 (88.9 %)	0.569
Alcohol (%)	3/12 (25.0 %)	4/15 (26.7 %)	7/27 (25.9 %)	1.00
Quit Line (%)	0/11	0/14	0/25	-
Other cessation program/Medication Use (%)	2/11 (18.2 %)	1/14 (7.1 %)	3/25 (12 %)	1.000
Infection Post DC (%)	1/11 (9.1 %)	4/15 (26.7 %)	5/26 (19.2 %)	0.614
ER visit (%)^b^	7/17 (41.2 %)	7/22 (31.8 %)	14/39 (35.9 %)	0.689
Hospital readmission (%)^b^	4/17 (23.5 %)	8/22 (36.4 %)	12/39 (30.8 %)	1.000

^*^Data analyzed using Fisher’s exact analysis

^a^Quit Line and other program/medication use was included in the week 1 outcome follow-up beginning with patient 18 and in the 3 month outcome follow-up with subject 4

^b^ER visitation and Hospital readmission data were compiled from self-report and medical records data

**Fig. 2:**
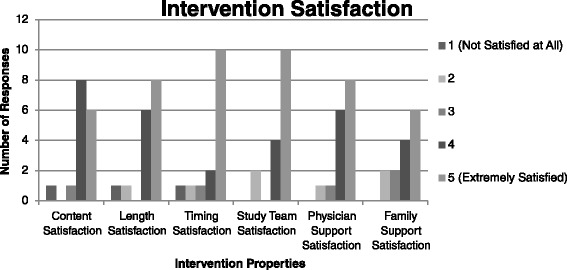
Subject Satisfaction with Intervention Procedures. Legend: Number analyzed: Intervention group *n* = 16

Subject satisfaction with the intervention was overall very high (Fig. 2). The average content satisfaction score was 4.13 (standard deviation [SD] = 1.02) on the Likert scale. The average length of intervention satisfaction score was 4.19 (SD = 1.17). The average timing satisfaction score was 4.27 (SD = 1.3). The average study team satisfaction score was 4.38 (SD = 1.02). The average physician support satisfaction score was 4.31 (SD = 0.87) and the average family support satisfaction score was 4.00 (SD = 1.02). Additionally, one subject expressed a desire to have more counseling sessions be part of the intervention protocol and another suggested that the sessions be longer. Others also suggested that there be more immediate action from the intervention, so that, instead of having to wait until they were discharged, they could actively engage in their tobacco cessation while still in the hospital (e.g., through the use of a nicotine patch or other medications). Lastly, some subjects suggested that the counseling session may be more effective if performed closer to their discharge from the hospital so that they were more likely to retain the information discussed during the encounter and not be as distracted by their current medical treatments.

At the week one follow-up, there were no statistically significant differences in the WSWS outcomes observed between the groups (Table 3). At the 3-month follow-up, the mean “thinking about food” score was significantly higher in the usual care group (Table 3). Additionally, the mean withdrawal assessment scores on the PANAS scale were not statistically significant at either follow-up time point (Table 4).

**Table 3:** Wisconsin Smoking Withdrawal Scale and Self-Efficacy Assessment (WSWS) mean scores at 1 week and 3 months

	1 week			3 months		
Scale	Intervention group avg. score (SD^a^) (*n* = 16)	Usual care avg. score (SD^a^) (*n* = 21)	*p*-value^*^	Intervention group avg. score (SD^a^) (*n* = 11)	Usual care avg. score (SD^a^) (*n* = 15)	*p*-value^*^
Anxiety	2.9 (1.4)	3.1 (1.2)	0.543	2.5 (1.3)	3.5 (1.4)	0.052
Impatience	2.6 (1.2)	3.00 (1.4)	0.321	2.4 (1.1)	2.8 (1.4)	0.397
Bothered by Negative Moods	2.8 (1.3)	2.9 (1.4)	0.734	2.5 (1.2)	2.9 (1.2)	0.518
Irritable or easily angered	2.6 (1.5)	2.9 (1.4)	0.625	2.5 (1.1)	2.6 (1.3)	0.768
Depressed or Sad	2.8 (1.3)	2.6 (1.2)	0.568	2.4 (1.4)	2.6 (1.1)	0.632
Hopeless or Discouraged	2.5 (1.2)	2.2 (1.0)	0.407	2 (1.1)	2.5 (1.1)	0.285
Difficulty Paying Attention	2.6 (1.4)	2.1 (0.9)	0.270	2.2 (1.3)	2.5 (1.2)	0.560
Difficulty Thinking Clearly	2.4 (1.2)	2.2 (1.0)	0.497	2.0 (1.1)	2.5 (1.1)	0.239
Think of Food a lot	2.5 (1.5)	3.2 (1.2)	0.137	2.1 (1.0)	3.1 (1.1)	0.020
Hunger	2.8 (1.1)	3.4 (1.2)	0.090	2.5 (1.1)	3.3 (1.2)	0.075
Craving	2.8 (1.3)	3.1 (1.1)	0.473	2.6 (1.4)	2.9 (1.4)	0.592
Self-Efficacy Assessment**	2.1 (0.9)	2.7 (1.2)	0.088	2.4 (1.2)	2.2 (0.9)	0.642

^a^SD = Standard deviation

^*^Data analyzed using *t*-tests

WSWS scores are measured on an agreement scale of 1 – 5; 1 = “strongly disagree”. 2 = “disagree”, 3 = “neutral” 4 = “agree”, 5 = “strongly agree”

**One subject did not respond to the self-efficacy statement in the intervention group; (*N* = 10)

**Table 4:** Positive and Negative Affect Schedule (PANAS) mean scores at 1 week and 3 months

	1 week			3 months		
Scale	Intervention group avg. score (SD^a^) (*n* = 16)	Usual care avg. score (SD^a^) (*n* = 21)	*p*-value^*^	Intervention group avg. score (SD^a^) (*n* = 11)	Usual care avg. score (SD^a^) (*n* = 15)	*p*-value^*^
Distressed	2.1 (1.4)	2.2 (1.3)	0.695	2.4 (1.5)	2.2 (1.3)	0.771
Upset	2.1 (1.3)	2.3 (1.3)	0.718	2.4 (1.6)	2.5 (1.4)	0.862
Strong	3.0 (1.2)	3.2 (1.2)	0.638	3.4 (1.2)	2.9 (1.4)	0.418
Enthusiastic	2.6 (1.3)	2.9 (1.2)	0.489	2.9 (1.2)	3.0 (1.3)	0.859
Irritable	2.4 (1.6)	2.5 (1.5)	0.869	2.5 (1.5)	2.4 (1.4)	0.925
Determined	3.8 (1.1)	3.2 (1.1)	0.134	3.7 (1.3)	3.1 (1.3)	0.269

^a^ *SD* standard deviation

^*^Data analyzed using *t*-tests

PANAS scores are measured on an agreement scale of 1 – 5; 1 = “strongly disagree”. 2 = “disagree”, 3 = “neutral” 4 = “agree”, 5 = “strongly agree”

## Discussion

In this study, aimed at examining the feasibility and acceptability of tobacco cessation interventions in hospitalized patients, we found that overall, while recruitment of subjects was achievable, retention in the study and collecting discharge swabs was a challenge in this group of patients. Subjects showed consistently low withdrawals symptom levels and only differed significantly in one WSWS scale measure and no PANAS scale measures. They also had high levels of satisfaction with the tobacco cessation intervention. For each measure of satisfaction, subjects reported an average Likert scale score of 4 or higher, out of a possible 5. Due to the high levels of satisfaction seen in our subjects upon administration of this intervention, we anticipate that interventions of this kind are acceptable to inpatients. However, close attention will need to be paid to put measures in place to minimize attrition and improve swab collection at discharge.

We also found that subject satisfaction with the intervention did not translate into cessation of tobacco use as we still observed low quit rates in each of the study groups. This low rate of tobacco cessation could also have influenced the low levels of withdrawal symptoms observed in the groups as well. There are a number of possible explanations for this finding. First, none of the subjects in either treatment or usual care group utilized free tobacco cessation services such as the Wisconsin Quit Line services. Barriers to use exist, such as the need to locate and call a number and allocate sufficient time to discuss a cessation plan with the Quit Line staff. Additionally, some subjects expressed a desire for more immediate action from the intervention instead of having to wait until they were discharged to put the tobacco cessation strategies discussed during the counseling session into practice. This suggests that the use of seamless referrals, through the electronic medical record of patients, to cessation programs and materials, initiated while they are still in the hospital, may be an important method to facilitate subject engagement with and adherence to a smoking cessation plan. This also may indicate that the level of counseling intervention was not high enough and subjects may benefit from more counseling sessions or longer counseling sessions. Referral systems of this kind have been shown to be effective and have been successfully implemented in hospital inpatient settings and should therefore be explored further as part of the cessation program [23]. Second, the timing of the intervention may also be important. Some subjects expressed that the counseling session may be more effective if performed closer to their discharge from the hospital so that they were less distracted by their hospital treatments and more likely to retain the information.

We found that participants in both groups had high rates of post discharge emergency room visits and hospital readmission, as well as high rates of new infection including pneumonia, surgical site infection, sinus infection, and urinary tract infection in the 3-month period of follow-up. Furthermore, approximately 21 % of our sample was colonized by *S.aureus* at baseline and one subject observed as negative at baseline became positive at discharge. The higher rates of infection at admission however, may have played a role in the rates of infection seen at and post discharge. Ultimately, these high rates of infection and healthcare utilization highlight the susceptibility of this population to infections and may also be useful in determining the effect size and sample size needed in a larger study of tobacco cessation as a tool to reduce HAIs.

Our study extends current literature in this area. Rigotti et al. undertook a prospective observational study of six hundred fifty adult smokers admitted to the medical and surgical services of a large urban teaching hospital and found that only 34 of 650 smokers (5.2 %) received nicotine replacement therapy (NRT) during their hospital stay [24]. The authors also note that NRT was typically used to lessen nicotine withdrawal symptoms without taking into account the patient’s ultimate tobacco cessation goals but did not offer suggestions as to why this may be. Ultimately, they concluded that the use of NRT should be increased in hospitalized smokers to decrease nicotine withdrawal symptoms and also encourage tobacco cessation [24]. A recent published study protocol describes a multi-center, randomized clinical effectiveness trial planned to be conducted at Kaiser Permanente Northwest and at Oregon Health & Science University (OHSU) hospitals in Portland, Oregon. It will recruit 900 hospital adult inpatients who smoke and will implement an intensive counseling intervention to examine cessation outcomes, with a primary outcome of self-reported 30-day smoking abstinence at 6 months post-randomization for intervention participants compared to usual care [25]. However, this study does not include assessment of healthcare-associated infection. Accordingly, our study sets the stage for a larger randomized trial to examine the impact of smoking cessation intervention on HAI and post discharge infections by first gathering critical data on the acceptability of such an intervention and feasibility in inpatient smokers as well as offering insight into the intervention methods that will be most successful.

Our study has several limitations. The sample size was small, restricting the ability to fully characterize potential tobacco cessation engagement and outcomes, as well as limiting the statistical power of our associations. Even with randomization, the usual care group showed much higher rates of infection at admission which may have influenced the rates of infections recorded at discharge and at each follow-up. There were also a high number of subjects lost to follow-up which could bias our observed results. Increasing sample size and limiting losses to follow-up may better randomize baseline infections between study groups and aid in distinguishing the possible effectiveness of the intervention and differences in outcomes. It was not possible in this study to use blinded reviewers for the follow-up assessments. Participation among those not interested in quitting was low, with many inpatients declining to participate. Many were simply not interested in quitting smoking at the time of recruitment or were too preoccupied with their current hospital stay to want to participate in a study. Accordingly, if the future intervention can more be seamlessly integrated into their hospital care, participation may increase. These limitations notwithstanding, our findings have implications for clinicians, infection preventionists and hospital personnel involved in efforts to prevent infections including HAI.

## Conclusions

In conclusion, our results suggest that a brief face to face behavioral tobacco cessation intervention is feasible and acceptable for inpatient smokers. Study efficacy could be improved by the addition of a seamless referral mechanism to cessation programs and materials while the patient is still in the hospital and scheduling outpatient counseling at the end of a patient’s stay. An important next step would be to evaluate the impact of this intervention on HAI and other infection rates in a large, adequately powered, randomized controlled trial.

## Ethics approval and consent to participate

The study protocol was approved by the Institutional Review Board at the University of Wisconsin School of Medicine and Public Health.

## Abbreviations

HAI: hospital-acquired infection
MRSA: methicillin resistant *Staphylococcus aureus*
MSSA: methicillin susceptible *Staphylococcus aureus*
NRT: nicotine replacement therapy
PANAS: positive and negative affect schedule
SD: standard deviation
WSWS: wisconsin smoking withdrawal scale
